# Geniposide and Chlorogenic Acid Combination Ameliorates Non-alcoholic Steatohepatitis Involving the Protection on the Gut Barrier Function in Mouse Induced by High-Fat Diet

**DOI:** 10.3389/fphar.2018.01399

**Published:** 2018-12-11

**Authors:** Jing-hua Peng, Jing Leng, Hua-jie Tian, Tao Yang, Yi Fang, Qin Feng, Yu Zhao, Yi-yang Hu

**Affiliations:** ^1^Institute of Liver diseases, Shuguang Hospital affiliated to Shanghai University of Traditional Chinese Medicine, Shanghai, China; ^2^Institute of Clinical Pharmacology, Shuguang Hospital affiliated to Shanghai University of Traditional Chinese Medicine, Shanghai, China; ^3^Key Laboratory of Liver and Kidney Diseases (Shanghai University of Traditional Chinese Medicine), Ministry of Education, Shanghai, China; ^4^Shanghai Key Laboratory of Traditional Chinese Clinical Medicine, Shanghai, China; ^5^Department of Cardiology, Cardiovascular Research Institute, Shuguang Hospital affiliated to Shanghai University of Traditional Chinese Medicine, Shanghai, China

**Keywords:** non-alcoholic fatty liver disease, non-alcoholic steatohepatitis, gut-liver axis, gut barrier function, geniposide, chlorogenic acid, complementary and alternative medicine

## Abstract

Gut-liver axis is increasingly recognized to be involved in the pathogenesis and progression of non-alcoholic fatty liver disease (NAFLD). The gut microbiota and intestinal permeability have been demonstrated to be the key players in the gut-liver cross talk in NAFLD. Geniposide and chlorogenic acid (GC) combination is derived from a traditional Chinese medicine, Qushi Huayu Decoction (QHD), which has been used in clinic for NAFLD treatment for decades in China and validated in multiple animal models of NAFLD. GC combination previously has been demonstrated to treat NAFLD via modulation on the gut microbiota composition. In the present study, the effects of GC combination on gut barrier function in NAFLD were evaluated, and QHD and sodium butyrate (NaB), the intestinal mucosa protectant, were used as positive control. The therapeutic effect of GC combination on NAFLD were confirmed by amelioration on non-alcoholic steatohepatitis (NASH) induced by high-fat diet (HFD) in mouse, which was comparable to that of QHD. Simultaneously, GC combination was found to reduce the signaling of gut-derived lipopolysaccharide (LPS) including hepatic LPS binding protein, Toll like receptor 4, interleukin-1β, tumor necrosis factor –α, and Kupffer cells infiltration. Furthermore, GC combination reduced LPS and D-lactate in plasma, restoring the colonic tight junction (TJ) expression and inhibited colonic TJs disassembly by down-regulation on RhoA/ROCK signaling in NASH induced by HFD. On the other hand, NASH was also alleviated in NaB group. The results of the present study suggested the important role of protection on gut barrier function in NAFLD treatment, which contributed to the therapeutic effects of GC combination on NASH.

## Introduction

Non-alcoholic fatty liver disease (NAFLD) is characterized by excessive hepatic steatosis (more than 5% hepatocytes) lack of secondary causes of hepatic fat accumulation such as alcohol consumption, long-term use of a steatogenic medication or monogenic hereditary disorder (Chalasani et al., 2018). The spectrum of histological manifestations of NAFLD includes simple steatosis, non-alcoholic steatohepatitis (NASH), cirrhosis and hepatocellular carcinoma (Torres et al., 2012), in which, NASH is the key step and relative to the higher motility in patients of chronic liver disease (Dulai et al., 2017). NAFLD has emerged as one of the most prevalent liver diseases in many countries since it is commonly associated with metabolic comorbidities such as obesity, type 2 diabetes, and dyslipidemia (Loomba and Sanyal, 2013). The mechanism of pathogenesis and progression of NAFLD is multifactorial and complex (Buzzetti et al., 2016). Among the multiple hits, the gut-liver axis is increasingly recognized to be implicated in NASH onset and progression. The gut microbiota and intestinal permeability have been demonstrated to be the key players in the gut-liver cross talk in NAFLD (Cani et al., 2007; Federico et al., 2016; Leung et al., 2016).

Geniposide and chlorogenic acid (GC) combination is derived from a traditional Chinese medicine, Qushi Huayu Decoction (QHD), consisting of five herbs including *Artemisia capillaries* Thunb., *Gardenia jasminoides* Ellis, *Polygonum cuspidatum* Sieb. et Zucc., *Curcuma longa* L., and *Hypericum japonicum* Thunb., which has been used in clinic for NAFLD treatment for decades in China and validated in multiple animal models of NAFLD (Feng et al., 2010, 2013, 2017; Yin et al., 2013). In our previous study, the principle active component of each Chinese medicinal herb presented in QHD were screened at different dosages by uniform design, and hepatic triglyceride (TG) was used as the screening index in NAFLD induced by high fat diet (HFD) in mice (Tang et al., 2013). The regression equation suggested that the formula consisting of geniposide from *G. jasminoides* Ellis (90 mg/kg per day) and chlorogenic acid from *A. capillaries* Thunb. (1.34 mg/kg per day) exhibited the most powerful inhibition on lipid deposition in NAFLD mice (Tang et al., 2013; Feng et al., 2017).

The mechanisms investigation has demonstrated that GC combination treated NAFLD via modulation on the structure of the gut microbiome (Feng et al., 2017). In the present study, the effects of GC combination on gut barrier function in NAFLD were evaluated to further identify the role of GC on gut-liver axis in NAFLD. QHD, as the mother drug of GC combination, was used as positive control for NAFLD treatment. Sodium butyrate (NaB) is a member of short chain fatty acids, which is the bacterial fermentation products and strengthen the gut barrier function (Zhu et al., 2015). In the present study, NaB was used as a positive control of intestinal mucosa protectant.

## Materials and Methods

### Plant Materials and Standards

Geniposide (IUPAC name: methyl (1S,4aS,7aS)-7-(hydroxymethyl)-1-[(2S,3R,4S,5S,6R)-3,4,5-trihydroxy-6-(hydroxymethyl)oxan-2-yl]oxy-1,4a,5,7a-tetrahydrocyclopenta[c]pyran-4-carboxylate, purity >98%) and chlorogenic acid (IUPAC name: (1S,3R,4R,5R)-3-[(E)-3-(3,4-dihydroxyphenyl)prop-2-enoyl]oxy-1,4,5-trihydroxycyclohexane-1-carboxylic acid, purity >98%) are commercial products of Shanghai Winherb Medical Technology Co., Ltd. (Shanghai, China).

The qualified commercial Chinese medicinal herbs presented in QHD (Table 1) were purchased from Shanghai Tongji Tang Pharmaceutical Co., Ltd. (Shanghai, China). Raw materials were authenticated macroscopically and microscopically according to The Pharmacopeia of the People's Republic of China (2015 Edition) and The Drug Standard of Ministry of Public Health of the People's Republic of China (1992 Edition). Their voucher specimens were deposited at Shuguang Hospital affiliated to Shanghai University of Traditional Chinese Medicine (Shanghai, China). The plant names have been checked against http://www.theplantlist.org database. The standards including geniposide, chlorogenic acid, resveratrol (IUPAC name: 5-[(E)-2-(4-hydroxyphenyl)ethenyl]benzene-1,3-diol) and polydatin (IUPAC name: (2S,3R,4S,5S,6R)-2-[3-hydroxy-5-[(E)-2-(4-hydroxyphenyl)ethenyl]phenoxy]-6-(hydroxymethyl)oxane-3,4,5-triol) were purchased from Shanghai R&D Center for Standardization of Chinese Medicines (Shanghai, China).

**Table 1:** Composition of QHD.

**Pharmaceutical name**	**Botanical name**	**Family and plant part use**	**Chinese name**	**Origin**	**% (w/w)**
Artemisiae scopariae herba	*Artemisia capillaris* Thunb.	Compositae; Above-ground parts, dried	Yinchen	Hebei Province, China	28.6
Polygoni cuspinati rhizome et radix	*Polygonum cuspidatum* Sieb. et Zucc.	Polygonaceae; root and rhizome, dried	Huzhang	Jiang su, Province, China	21.4
Curcumae longae rhizome	*Curcuma longa* L.	Zingiberaceae; root, dried	Jianghuang	Si chuan, Province, China	14.3
Gardeniae fructus	*Gardenia jasminoides* Ellis	Rubiaceae; ripe fruit, dried	Zhizi	Jiang xi, Province, China	14.3
^*^Herba hyperici japonici	*Hypericum japonicum* Thunb.	Clusiaceae; whole plant, dried	Di'ercao	Hu nan, Province, China	21.4

* *The botanical names of Di'ercao is from The Drug Standard of Ministry of Public Health of the People's Republic of China (1992 Edition), the others are from The Pharmacopeia of the People's Republic of China (2015 Edition). The botanical names have been updated with www.theplantlist.org*.

### QHD Preparation

According to the patent of QHD preparation method (the State Intellectual Property Office of China, patent NO. ZL200610009140.0.0), Gardeniae fructus and Herba hyperici japonica were extracted with water, while the others were extracted with ethanol. The aqueous and the ethanol extracts were mixed and condensed to the density of 0.93 g crude herb/ml, and stored at −80°C until further use.

### Ultra-High Performance Liquid Chromatography—Mass Spectrometry Analysis

The standards including geniposide, chlorogenic acid, resveratrol, and polydatin were used as the quality control of QHD and the fingerprint spectrum was established by ultra-high performance liquid chromatography—mass spectrometry (UHPLC-MS) method. The chromatographic profile of QHD was shown in Supplementary Figure 1. The analysis was performed with a UHPLC-Q/Exactive system (Thermo, San Jose, CA, USA) equipped with a quaternary gradient pump, an autosampler and high-resolution mass spectrometry detector. The components were eluted with a gradient system consisting of acetonitrile (I) and water (II) in gradient (time, min/II%: 0/95, 18/5). The mass detector molecular weight was set in the range of 100~1,000 Da. The contents of chlorogenic acid, geniposide, polydatin, and resveratrol were 5.61, 6.17, 14.16, and 13.11 mg/ml in QHD, respectively (Supplementary Figure 1).

### Animals and Treatment

Male C57BL/6 mice, 8-week old, were purchased from Shanghai Experimental Animal Center of Chinese Academy of Sciences (Shanghai, China). They were maintained in Shanghai University of Traditional Chinese Medicine, Division of Animal Resources. All animal studies were approved by the animal studies ethics committee of Shanghai University of Traditional Chinese Medicine.

Mice were randomly divided into control (control diet, D12450B, 10% kcal from fat, Research Diets Inc. NJ, US) (*n* = 9), high fat diet (HFD, D12492, 60% kcal from fat, Research Diets Inc. NJ, US) (*n* = 9), HFD plus GC (geniposide 90 mg/kg per day and chlorogenic acid 1.34 mg/kg per day) (*n* = 9), HFD plus QHD (10 ml/kg per day) (*n* = 9), and HFD plus NaB (200 mg/kg per day, Sigma-Aldrich, US) group (*n* = 9) (Zhou et al., 2017). GC combination and NaB were dissolved in double distilled water (DDW) and prepared daily. After 12 weeks on the respective diets, mice were administrated with drugs intragastrically. Mice in control and HFD group received equal volume of DDW. Food intake and body weight were checked and recorded weekly. At the end of 16th week, mice were harvested and the plasma, liver and colon tissue were collected for assay.

### Histopathology

To observe the pathological changes, liver and colon tissue were fixed in 10% neutral formalin and embedded in paraffin. Sections (4 μm thick) were stained with hematoxylin and eosin (H&E) (Nanjing Jiancheng Institute of Bio Engineering, Inc., Nanjing, China) and examined under a light microscope (Olympus Medical Systems, Tokyo, Japan). Histological liver damage was evaluated via NAFLD activity score (NAS) which includes steatosis, lobular inflammation, and hepatocellular ballooning (Table 2; Kleiner et al., 2005). NAS of higher than 5 is correlated with a diagnosis of NASH. NAS of < 3 is diagnosed as “not NASH” (Kleiner et al., 2005).

**Table 2:** NAFLD activity score (NAS) system.

**Item**	**Definition**	**Score**
Steatosis	<5%	0
	5–33%	1
	>33–66%	2
	>66%	3
Lobular inflammation	No foci	0
	<2 foci per 200 × field	1
	2–4 foci per 200 × field	2
	>4 foci per 200 × field	3
Ballooning	None	0
	Few balloon cells	1
	Many cells/prominent ballooning	2

*NAS is the unweighted sum of steatosis, lobular inflammation, and hepatocellular ballooning scores. NAS of >5 correlated with a diagnosis of NASH, and biopsies with scores of < 3 were diagnosed as “not NASH” (Kleiner et al., 2005)*.

To observe the lipid deposition, liver tissue were embedded in Optimal Cutting Temperature medium (OCT, Sakura Finetek, Torrance, CA) and snap frozen in liquid nitrogen. The sections with 10 μm thick were stained with oil red (Sigma, MO, US) to visualize the lipid drop within the hepatocyte.

### Biochemical Assays

The activity of alanine aminotransferase (ALT) in plasma was determined with the biochemical assay kit (Nanjing Jiancheng Bioengineering Institute, Nanjing, People's Republic of China). Liver tissue (200 mg) was homogenized in 3 ml of ethanol-acetone mixture (1:1 in volume). The total hepatic TG was extracted in the medium at 4°C, overnight. After being centrifuged at 1,000 g for 20 min at 4°C, the supernatant was removed for the TG assay according to the instruction of the commercial kit (Dongou Bioengineering, Zhejiang, People's Republic of China).

### Enzyme Linked Immunosorbent Assay

Commercial enzyme linked immunosorbent assay (ELISA) kits were used for assay of hepatic lipopolysaccharide binding protein (LBP) (CKM043, Cell sciences Inc., MA, US), interleukin (IL)-1β (MBS036031, MyBioSource, Inc., CA, US), and tumor necrosis factor (TNF)-α (MBS49535, MyBioSource, Inc., CA, US) according to the manufacturer's instruction. Liver tissue (50 mg) was homogenized in 500 μl of phosphate buffer saline (PBS, PH 7.0–7.2). The homogenates was centrifuged at 1,500 g for 15 min at 4°C. Collect the supernatant for assay immediately.

### Assay for Lipopolysaccharide and D-Lactate

Blood was collected in a pyrogen-free, heparin-pretreated tube and centrifuged at 500 g for 15 min at 4°C. The plasma was removed immediately for lipopolysaccharide (LPS) analysis with the Pyrochrome Limulus Amebocyte Lysate kit (Associates of Cape Cod, East Falmouth, MA), according to the manufacturer's instructions. D-Lactic acid in plasma was determined by PicoProbe D-Lactate Assay Kit (Fluorometric) (ab174096, Abcam, MA, US) according to the manufacturer's instructions.

### Immunofluorescence Staining

Tissue was embedded in OCT medium (Sakura Finetek, Torrance, CA) and snap frozen in liquid nitrogen. The immunofluorescence staining protocol has been described previously (Cao et al., 2017). Briefly, the cryostat sections with 8 μm thick were fixed in ice cold acetone for 15 min, rinsing in PBS, 5 min, 3 times, permeabilization in 0.2% Triton X-100 (diluted in PBS) for 5 min. After rinsing in PBS for 5 min, sections were blocked with 3% horse serum for 1 h at room temperature and then incubated with primary antibodies (Table 3) at 4°C overnight. The sections were rinsed in PBS for 5 min, 3 times, and then incubated with the secondary antibodies (Table 3) for 30 min at room temperature in dark. Sections were finally mounted in VECTASHIELD mounting medium with DAPI (H-1200, VECTOR LABORATORIES, INC. CA, US). The sections were observed under laser scanning confocal microscope (OLYMPUS-FV10i, Olympus Corporation, Tokyo, Japan).

**Table 3:** Antibodies for western blotting and immunofluorescence staining.

**Antibody**	**Manufacturer catalog#**	**Species**	**Dilution**	
			**Western blotting**	**Immunofluorescence staining**
F4/80	Abcam, ab6640	Rabbit	–	1:100
TLR4	Abcam, ab13556	Rabbit	1:500	–
ZO-1	Thermo Fisher, 40-2200	Rabbit	1:500	1:100
Occludin	Thermo Fisher, 40-4700	Rabbit	1:1,000	1:250
Claudin-1	Thermo Fisher, 71-7800	Rabbit	1:200	1:100
MLC2	Cell signaling Technology, 3672	Rabbit	1:1,000	–
Phosphor-MLC2 (Ser19)	Cell signaling Technology, 3675	Mouse	1:1,000	–
MYPT1	Cell signaling Technology, 2634	Rabbit	1:1,000	–
Phospho-MYPT1 (Thr696)	Cell Signaling Technology, 5163S	Rabbit	1:1,000	–
β-actin	Proteintech, 66009-1-lg	Mouse	1:5,000	–
Anti-rabbit IgG (DyLight^TM^ 680 Conjugate)	Cell Signaling Technology, 5366	Goat	1:10,000	–
Anti-mouse IgG (H+L) (DyLight 800 4X PEG Conjugate)	Cell Signaling Technology, 5257	Goat	1:10,000	–
Anti-rabbit IgG H&L (Cy3®)	Abcam, ab6939	Goat	–	1:1,000
Anti-rabbit IgG H&L (Alexa Fluor® 488)	Abcam, ab150077	Goat	–	1:1,000

### Western Blot Analysis

As described previously (Peng et al., 2008; Peng J. H. et al., 2009), the total protein was extracted from liver and colon tissues, and was determined with a bicinchoninic acid protein concentration assay kit (Thermo Fisher Scientific, MA, US). Western blot analysis was performed to evaluate the protein expression of hepatic Toll-like receptor (TLR) 4 and intestinal zonula occludens-1 (ZO-1), Occludin, Claudin-1, myosin light chain (MLC) 2, phosphorylated MLC2 (p-MLC2), myosin phosphatase target subunit (MYPT) 1, and phosphorylated MYPT1 (p-MYPT1). Equal amounts of proteins were loaded and separated by 8–15% SDS-polyacrylamide gel electrophoresis under non-reducing and denaturing conditions and then transferred onto polyvinylidene difluoride membranes (Millipore, MA, US). The membranes were blocked with 5% bovine serum albumin (Sigma, MO, US) for 1 h and then incubated with primary antibodies overnight at 4°C (Table 3).The membrane were then incubated with the corresponding secondary antibodies (Table 3) at room temperature for 1 h and finally were scanned with the Odyssey quantitative western blot near-infrared system (LI-COR Biosciences, NE, US). The density of bands was analyzed with Odyssey application software version 3.0 (LI-COR Biosciences, NE, US). The relative expression levels of proteins were corrected by β-actin. While the relative expression levels of p-MLC2 and p-MYPT1 were corrected by MLC2 and MYPT1, respectively. The data represent the fold changes relative to that in control group.

### Assay for Activity of Rho-Associated Kinase, Active, and Total RhoA

Rho-associated kinase (ROCK) activity in colon tissue was detected by ROCK activity assay kit (CSA001, Millilpore, MA, US). The active and total RhoA in colon tissue was detected by G-LISA RhoA activation assay biochem kit (BK124, Cytoskeleton, Inc., CO, US), and total RhoA ELISA kit (BK150, Cytoskeleton, Inc., CO, US), respectively. The colon tissue sample was prepared according to the instruction of these commercial kits.

### Statistical Analysis

Results were expressed as mean ± standard deviation. For comparison of more than two groups, the statistical significance was calculated by one-way analysis of variance (ANOVA) followed by Bonferroni's Multiple Comparison Test. The non-parametric data were analyzed by ANOVA followed by Kruskal-Wallis *H*-test. Significance was accepted at the level of *P* < 0.05.

## Results

### GC Combination Ameliorated Liver Injury and Lipid Deposition in NAFLD Induced by HFD

There is no statistical difference in the weekly food intake of each mouse among groups. At the end of 16th week, the body weight of mice in HFD group increased obviously comparing to that in control group. With treatment of GC combination, QHD, or NaB, the body weight decreased (Table 4).

**Table 4:** Food intake and body weight at the end of 16th week.

**Group**	***n***	**Weekly food intake (g/mouse)**	**Body weight (g)**
C	9	20.07 ± 6.11	29.98 ± 1.68
HFD	9	22.46 ± 6.48	47.57 ± 3.12^**^
GC	9	19.95 ± 5.64	42.33 ± 3.43^#^
QHD	9	20.05 ± 6.02	42.31 ± 3.62^#^
NaB	9	21.80 ± 5.93	44.22 ± 3.43^#^

Data were expressed as mean ± standard deviation.

** p < 0.01, vs. C,

# *p < 0.05, vs. HFD. C, control, HFD, high-fat diet, GC, geniposide and chlorogenic acid combination, QHD, Qushi Huayu Decoction, NaB, sodium butyrate*.

**Figure 1:**
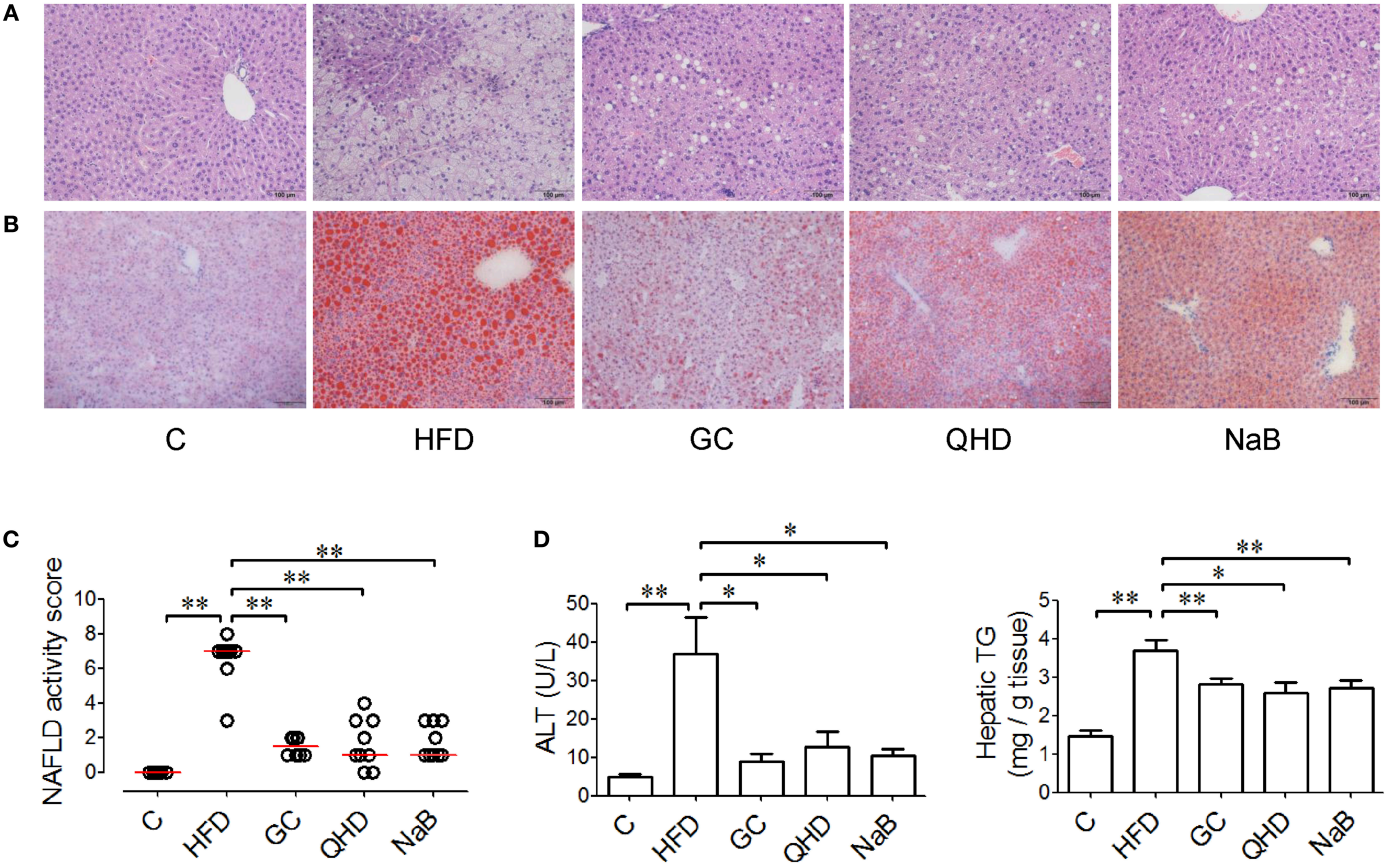
Effect of GC combination on liver injury induced by HFD. **(A)** Hematoxylin-eosin staining for liver sections (200 times of magnification). **(B)** Oil red staining (for visualization of the lipid deposition) for liver sections (200 times of magnification). **(C)** NAS. Data represents the median and individual NAS in each group. **(D)** ALT level in plasma and hepatic TG. C, control, HFD, high-fat diet, GC, geniposide and chlorogenic acid combination, QHD, Qushi Huayu Decoction, NaB, sodium butyrate, NAFLD, non-alcoholic fatty liver disease, NAS, NAFLD activity score. ^*^P < 0.05, ^**^P < 0.01.

After 16-week feeding with HFD, steatosis and ballooning of hepatocytes, and inflammatory cells infiltration was observed in lobules (Figures 1A,B). Macrovesicular steatosis was found mostly in centrilobular regions. As visualized in Oil red O staining, lipid vacuoles occupied much of the cytoplasm and the nucleus and other organelles were pushed to the periphery of the hepatocytes. Ballooning degenerated hepatocytes characterized by bloated and rarefied cytoplasm were common. The inflammatory cells were found around the necrotic hepatocytes. The median of NAS of mice in HFD group was higher than 5, which correlated to diagnosis of NASH (Figure 1C).

With treatment of GC combination or QHD, the steatosis of hepatocytes was ameliorated and rarer ballooning degeneration and inflammatory infiltration was observed (Figures 1A,B). NAS of mice in GC group decreased (*P* < 0.01) comparing to that in HFD group and was comparable to that in QHD group (Figure 1C).

Consistent to the pathological changes in the liver tissue, elevated ALT in plasma (*P* < 0.01) and hepatic TG content (*P* < 0.01) was detected in mice of HFD group. While, with treatment of GC combination, plasmic ALT (*P* < 0.05), and hepatic TG (*P* < 0.01) decreased comparing to that in HFD group and was comparable to that in QHD group (Figure 1D).

In the NaB control group, plasmic ALT (*P* < 0.05), hepatic TG content (*P* < 0.01), and pathological changes (NAS, *P* < 0.01) were all alleviated comparing to that in HFD group and the therapeutic effects of NaB were comparable to that of GC combination or QHD (Figure 1).

### GC Combination Decreased Hepatic Cytokines Production Mediated by Kupffer Cells and Endotoxin Receptors in NAFLD Induced by HFD

**Figure 2:**
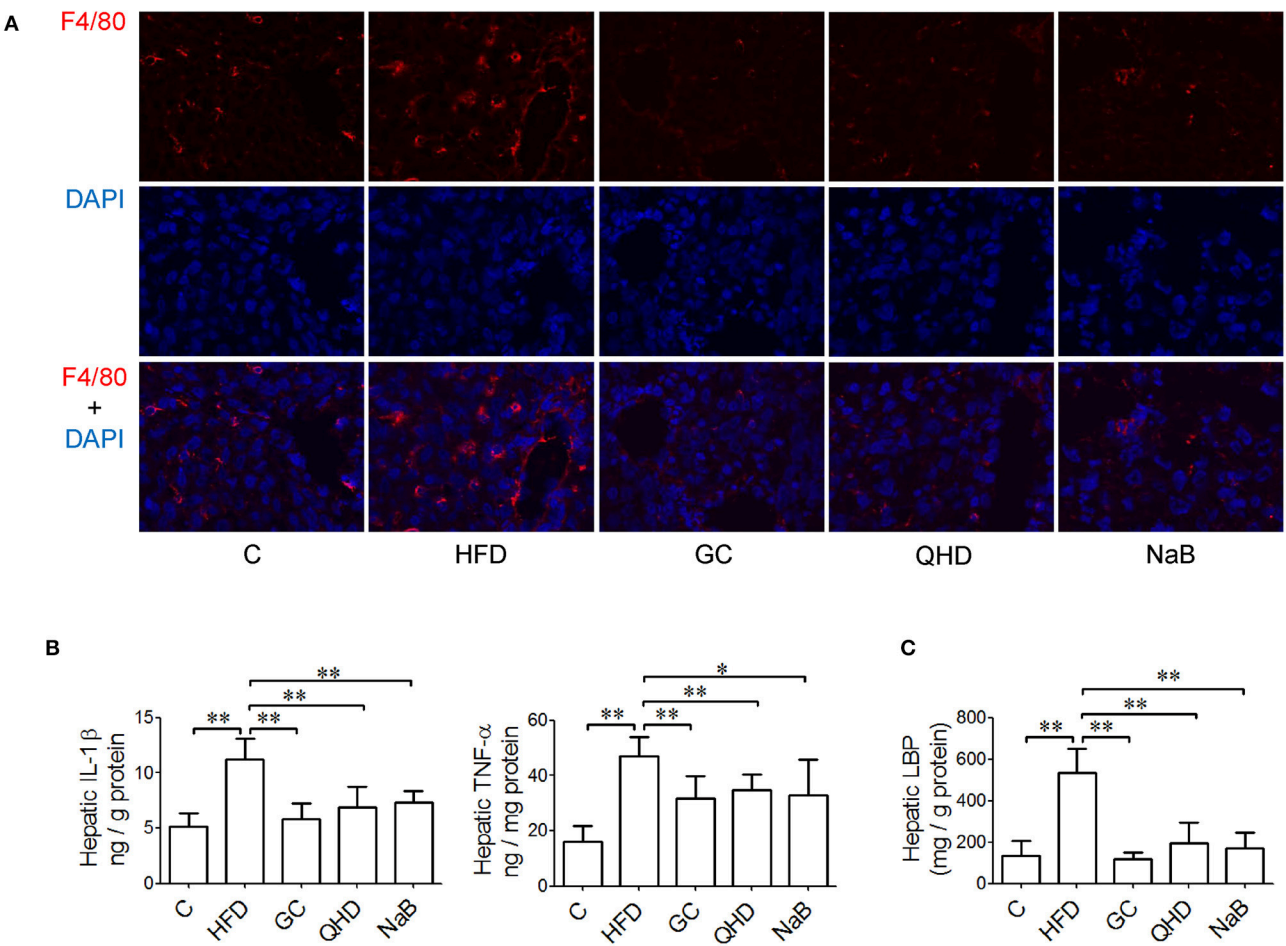
Effects of GC combination on hepatic Kupffer cells infiltration, cytokines production and LBP in NAFLD induced by HFD. **(A)** Immunofluorescence Staining for F4/80 in liver tissue (600 times of magnification). **(B)** Hepatic IL-1β and TNF-α content. **(C)** Hepatic LBP content. C, control, HFD, high-fat diet, GC, geniposide and chlorogenic acid combination, QHD, Qushi Huayu Decoction, NaB, sodium butyrate, LBP, lipopolysaccharide binding protein, NAFLD, non-alcoholic fatty liver disease. ^*^P < 0.05, ^**^P < 0.01.

**Figure 3:**
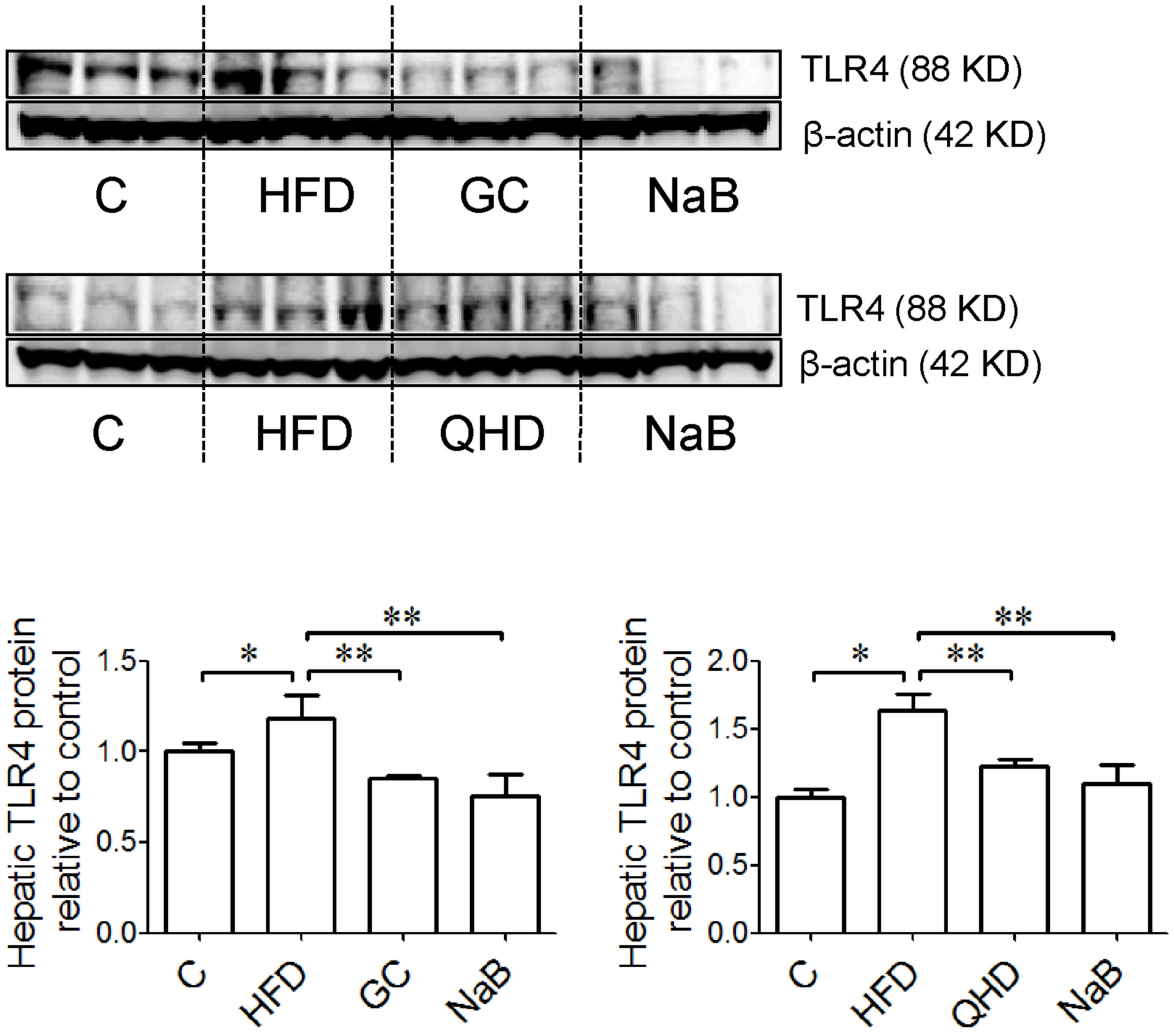
Effects of GC combination on hepatic TLR4 expression in NAFLD induced by HFD. TLR4 protein was detected by western-blot. The relative expression levels of proteins were corrected by β-actin. The data represents the fold changes relative to that in control group. C, control, HFD, high-fat diet, GC, geniposide and chlorogenic acid combination, QHD, Qushi Huayu Decoction, NaB, sodium butyrate, TLR4, Toll like receptor 4, NAFLD, non-alcoholic fatty liver disease. ^*^P < 0.05, ^**^P < 0.01.

In the immunofluorescence staining sections, the stronger positive staining of F4/80, the biomarker of kupffer cells (KCs), were observed in the liver tissue of mice fed with HFD (Figure 2A). Consistently, with HFD feeding, the hepatic TNF-α (*P* < 0.01) and IL-1β (*P* < 0.01) increased obviously (Figure 2B) accompanied with more severe pathological changes in liver tissue. The endotoxin receptors, including LBP (*P* < 0.01) and TLR4 (*P* < 0.05) were both high-expressed in the liver tissue of mice in HFD group (Figures 2C, 3).

In GC and QHD group, rarer positive staining of F4/80 was observed in the liver tissue. Meanwhile, the hepatic TNF-α (*P* < 0.01) and IL-1β (*P* < 0.01) decreased in GC group comparing to that in HFD group and was comparable to that in QHD group, as well as the hepatic LBP content (GC vs. HFD, *P* < 0.01, QHD vs. HFD, *P* < 0.01) (Figure 2) and TLR4 expression (GC vs. HFD, *P* < 0.01, QHD vs. HFD, *P* < 0.01) (Figure 3).

In NaB control group, the positive staining of F4/80, hepatic TNF-α (*P* < 0.05) and IL-1β (*P* < 0.01), LBP content (*P* < 0.01) (Figure 2), and TLR4 expression (*P* < 0.01) (Figure 3) were all decreased comparing to that in HFD group.

### GC Combination Ameliorated Gut-Leakage and Restored Colonic Tight Junctions Expression in NAFLD Induced by HFD

**Figure 4:**
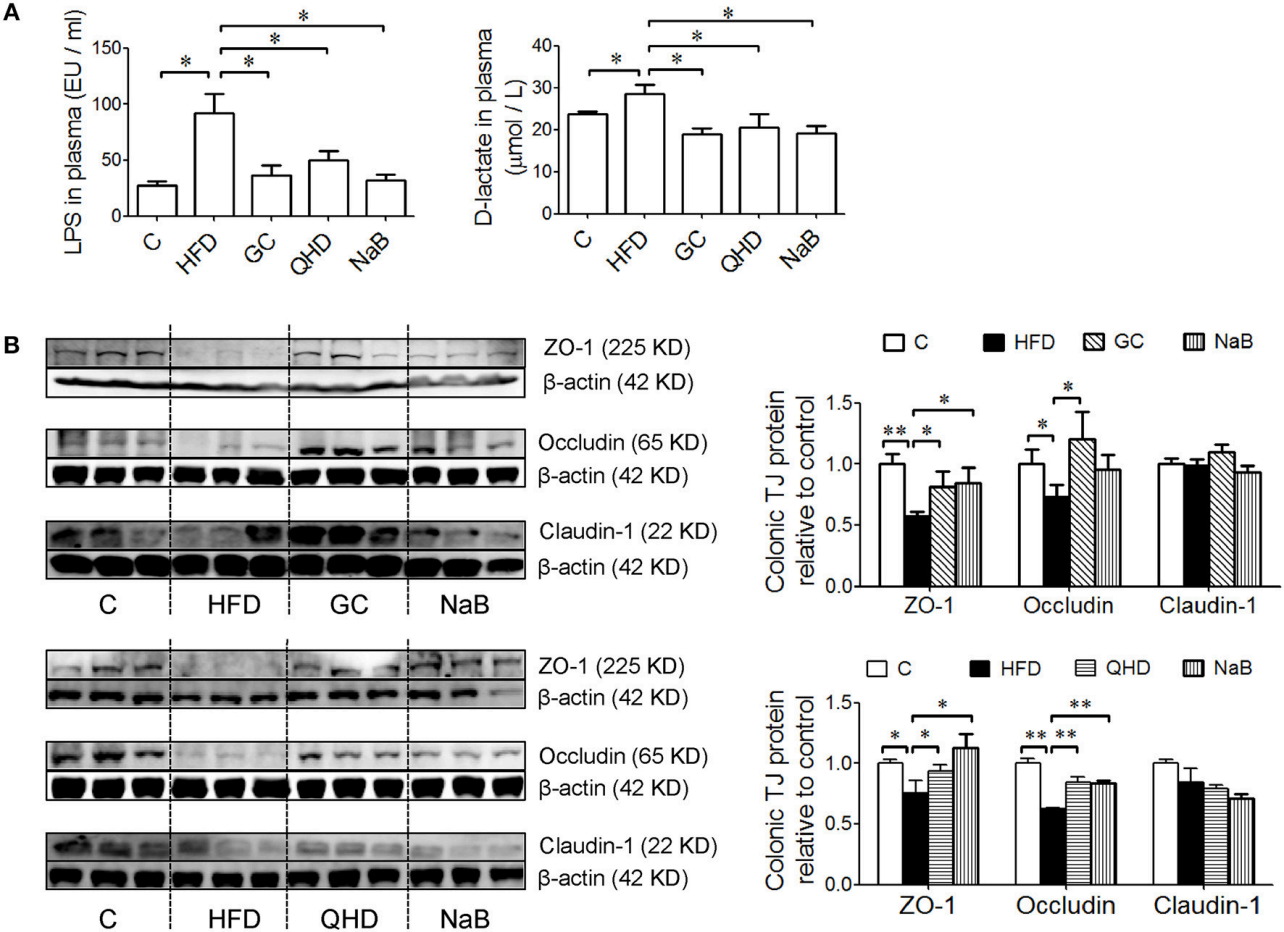
Effects of GC combination on LPS and D-lactate in plasma and tight junction protein expression in colon tissue in NAFLD induced by HFD. **(A)** LPS and D-lactate in plasma. **(B)** Protein expression of tight junction (ZO-1, Occludin and Claudin-1) in colon tissue detected by western-blot. The relative expression levels of proteins were corrected by β-actin. The data represents the fold changes relative to that in control group. C, control, HFD, high-fat diet, GC, geniposide and chlorogenic acid combination, QHD, Qushi Huayu Decoction, NaB, sodium butyrate, LPS, lipopolysaccharide, NAFLD, non-alcoholic fatty liver disease. ^*^P < 0.05, ^**^P < 0.01.

**Figure 5:**
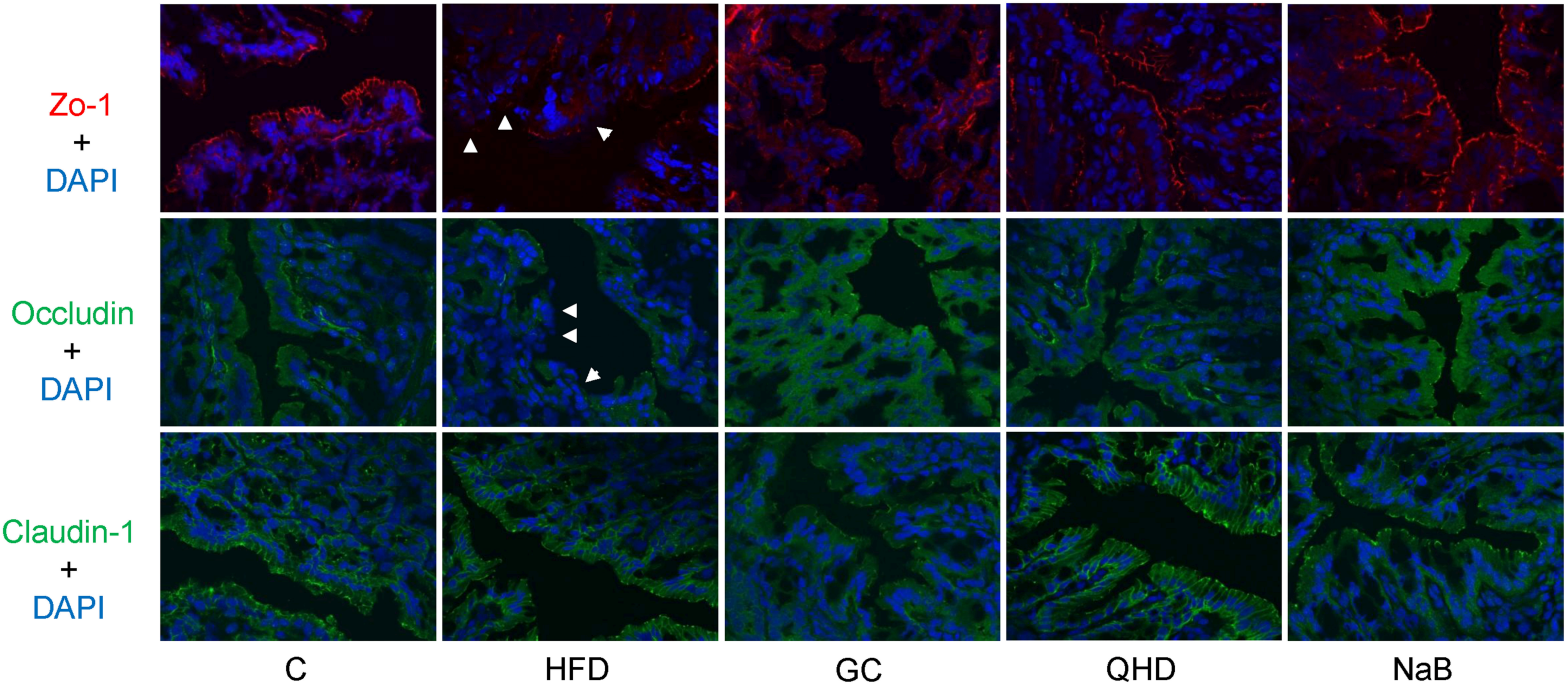
Immunofluorescence staining of tight junction (ZO-1, Occludin and Claudin-1) in colon. White arrows point out the tight junction disruption (600 times of magnification). C, control, HFD, high-fat diet, GC, geniposide and chlorogenic acid combination, QHD, Qushi Huayu Decoction, NaB, sodium butyrate.

Levels of LPS (*P* < 0.05) and D-lactate (*P* < 0.05) in plasma increased in HFD group, which indicated the intestinal permeability increased in the HFD-feeding mice (Figure 4A). Furthermore, the protein expression of tight junctions (TJs) including ZO-1 (*P* < 0.05) and Occludin (*P* < 0.01) was down-regulated in the colon of mice in HFD group (Figure 4B). As visualized in the immunofluorescence staining sections, ZO-1 and Occludin was disrupted and discontinuous in HFD group (Figure 5).

With treatment of GC combination, LPS (*P* < 0.05) and D-lactate (*P* < 0.01) in plasma decreased comparing to that in HFD group and was comparable to that in QHD-treated mice (Figure 4A). The protein expression of ZO-1 (GC vs. HFD, *P* < 0.01, QHD vs. HFD, *P* < 0.05) and Occludin (GC vs. HFD, *P* < 0.05, QHD vs. HFD, *P* < 0.01) was restored in GC and QHD group comparing to that in HFD group (Figure 4B). The visualization of ZO-1 and Occludin in colon tissue showed that complete and stronger positive staining in GC and QHD group (Figure 5).

As expected, with the treatment of intestinal mucosal protective agent, NaB, the levels of LPS (*P* < 0.05) and D-lactate (*P* < 0.01) in plasma were reduced comparing to that in HFD group (Figure 4). The protein expression of ZO-1 (*P* < 0.05) and Occludin (*P* < 0.01) was restored (Figure 4). And as visualized in the immunofluorescence staining of TJs, ZO-1 and Occludin in colon tissue was stronger positive stained in NaB group (Figure 5).

There was no significant difference of the protein expression of Claudin-1 among the groups.

### GC Combination Down-Regulated Colonic RhoA/ROCK Signaling in NAFLD Induced by HFD

**Figure 6:**
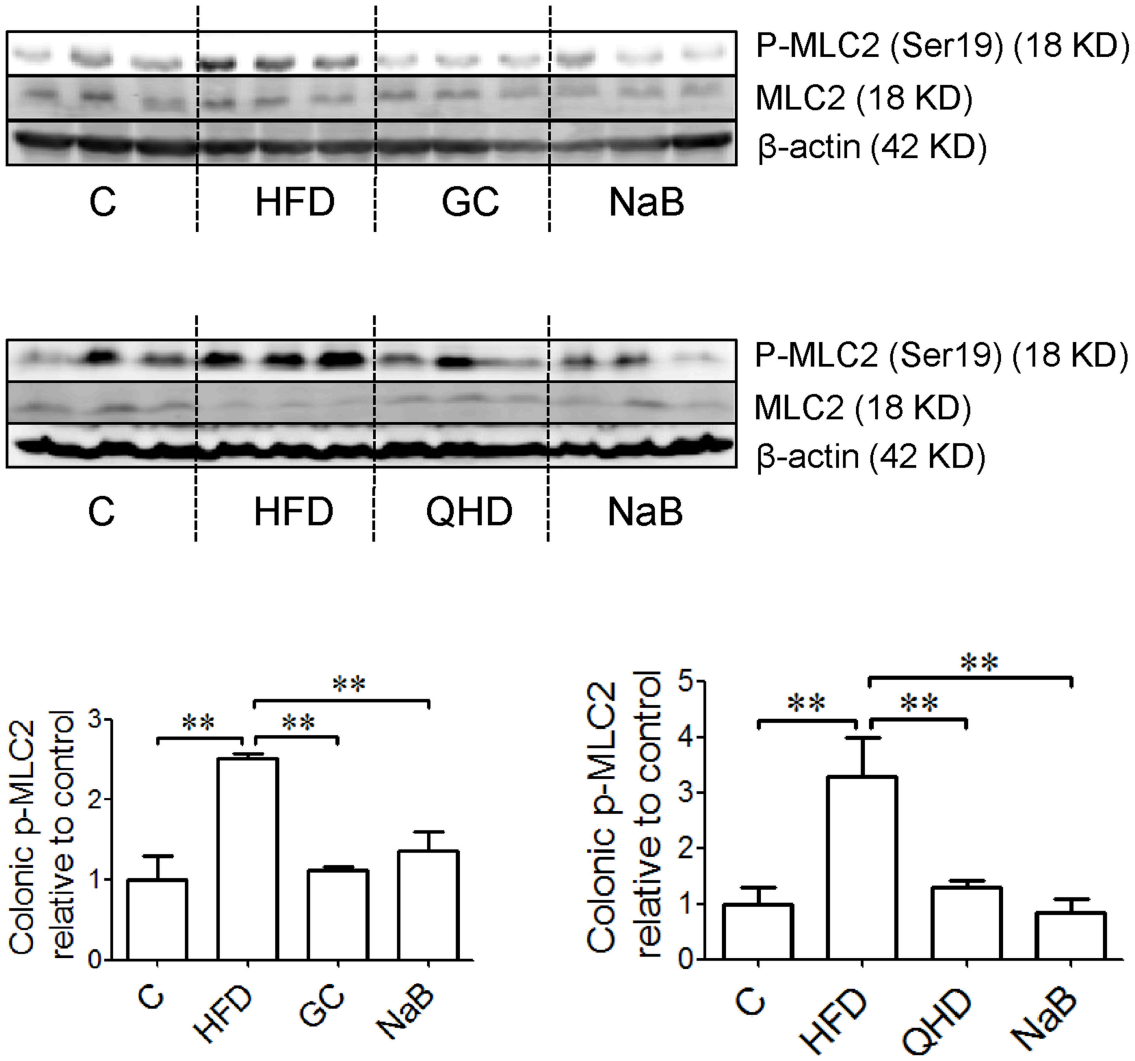
Effects of GC combination on phosphorylation of MLC2 in colon tissue in NAFLD induced by HFD. p-MLC2 was detected by western-blot. The relative expression levels of proteins were corrected by MLC2. The data represents the fold changes relative to that in control group. C, control, HFD, high-fat diet, GC, geniposide and chlorogenic acid combination, QHD, Qushi Huayu Decoction, NaB, sodium butyrate, p-MLC2, phosphorylated myosin light chain 2, NAFLD, non-alcoholic fatty liver disease. ^**^P < 0.01.

**Figure 7:**
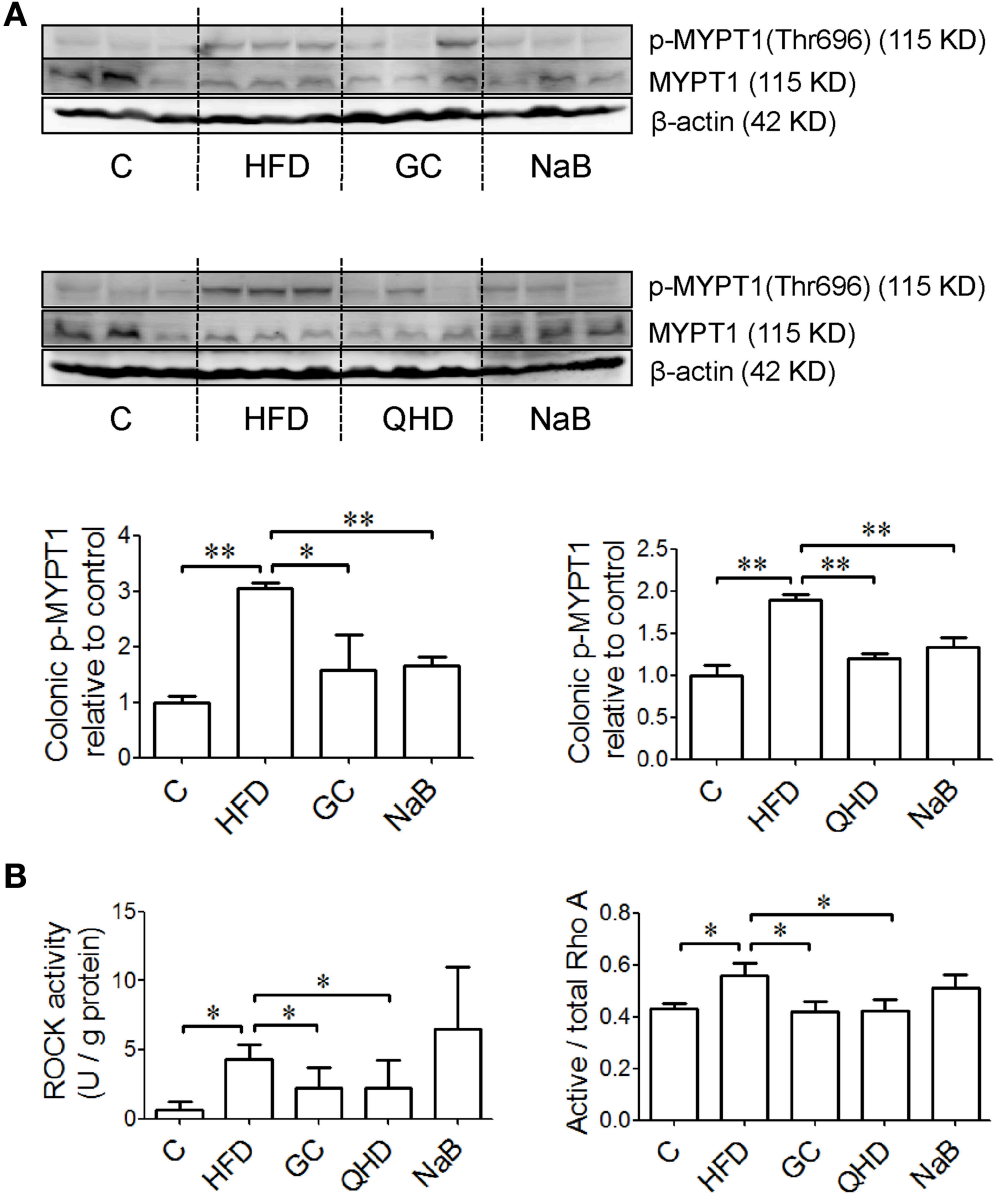
Effects of GC combination on phosphorylation of MYPT1, ROCK activity and ratio of active RhoA in colon tissue in NAFLD induced by HFD. **(A)** p-MYPT1 was detected by western-blot. The relative expression levels of proteins were corrected by MYPT1. The data represents the fold changes relative to that in control group. **(B)** ROCK activity and ratio of active RhoA in colon tissue. C, control, HFD, high-fat diet, GC, geniposide and chlorogenic acid combination, QHD, Qushi Huayu Decoction, NaB, sodium butyrate, p-MYPT, phosphorylated myosin phosphatase target subunit, ROCK, Rho-associated kinase, NAFLD, non-alcoholic fatty liver disease.^*^P < 0.05, ^**^P < 0.01.

The protein expression of p-MLC2 (Ser19) and p-MYPT1 (Thr696) increased in the colon tissue in HFD-feeding mice, while the MLC2 and MYPT1 protein expression did not changed obviously among groups. It was suggested that the phosphorylation of MLC2 at Ser19 (*P* < 0.05) (Figure 6) and MYPT1 at Thr696 (*P* < 0.01) (Figure 7A) was up-regulated in the colon tissue in HFD-feeding mice. While in GC or QHD-treated mice, the phosphorylation of MLC2 (GC vs. HFD, *P* < 0.01, QHD vs. HFD, *P* < 0.05) (Figure 6) and MYPT1 (GC vs. HFD, *P* < 0.05, QHD vs. HFD, *P* < 0.01) (Figure 7A) in the colon tissue was down-regulated.

The ratio of active RhoA (*P* < 0.05) and ROCK activity (*P* < 0.05) increased in the colon tissue of the mice fed with HFD. While, with treatment of GC or QHD, the colonic ratio of active RhoA (GC vs. HFD, *P* < 0.05, QHD vs. HFD, *P* < 0.05) and ROCK activity (GC vs. HFD, *P* < 0.05; QHD vs. HFD, *P* < 0.05) was down-regulated and there's no statistic difference between GC and QHD group. NaB treatment down-regulated the phosphorylation of MLC2 (*P* < 0.05) and MYPT1 (*P* < 0.01), but did not affect the active RhoA radio and ROCK activity in the colon tissue comparing to that in HFD group (Figure 7B).

## Discussion

### Gut-Derived LPS in NAFLD

Gut and liver have multiple levels of associated interdependence. They are embryologically linked, since the liver budding directly from the foregut during development. In anatomy, portal system transports the blood deriving from intestine to liver, which contains not only nutrients, but also the pathogen-associated molecular patterns including LPS, bacterial DNA, peptidoglycans and, in some cases, intact bacteria (Chassaing et al., 2014). Obese individuals and animals fed with HFD exhibit a change in gut microbiota composition as well as a 2 to 3-fold increase in blood LPS levels (Cani et al., 2008). Bacterial overgrowth in the small intestine was found in NAFLD patients and serum TNF-α level in NASH patients increased obviously (Wigg et al., 2001). On the other hand, intestinal permeability is different between simple steatosis and NASH patients, which indicated gut-derived factors including LPS, contribute to the progression of NAFLD through the leaky gut barrier (Farhadi et al., 2008).

KCs, the resident macrophages in the liver, are the major target cells of LPS and the primary cellular source of TNF-α in LPS stimulation. LPS signaling depends on a series of endotoxin receptors. LBP, produced mostly by hepatocytes (Su et al., 1994), binds to the lipid A portion of LPS with high affinity in the circulation and transfers the individual LPS molecules to the receptors on the surface of target cells (Gioannini and Weiss, 2007). TLR4 has been demonstrated to be the specific receptor of LPS from Gram-negative bacteria (Uesugi et al., 2001; Su, 2002) and delivers the LPS signal in myeloid differentiation factor 88 (MyD88) -dependent or MyD88-independent way and ultimately active KCs.

Consistent to the previous studies, our data showed that 16-week HFD induced NASH in mice and endotoxaemia appeared simultaneously. The hepatic content of LBP and protein expression of TLR4 was increased with HFD-feeding, as well as KCs infiltration and proinflammatory cytokines production in liver tissue. These data indicated that gut-derived LPS and the subsequent signaling participated in the pathogenesis of NASH.

**Figure 8:**
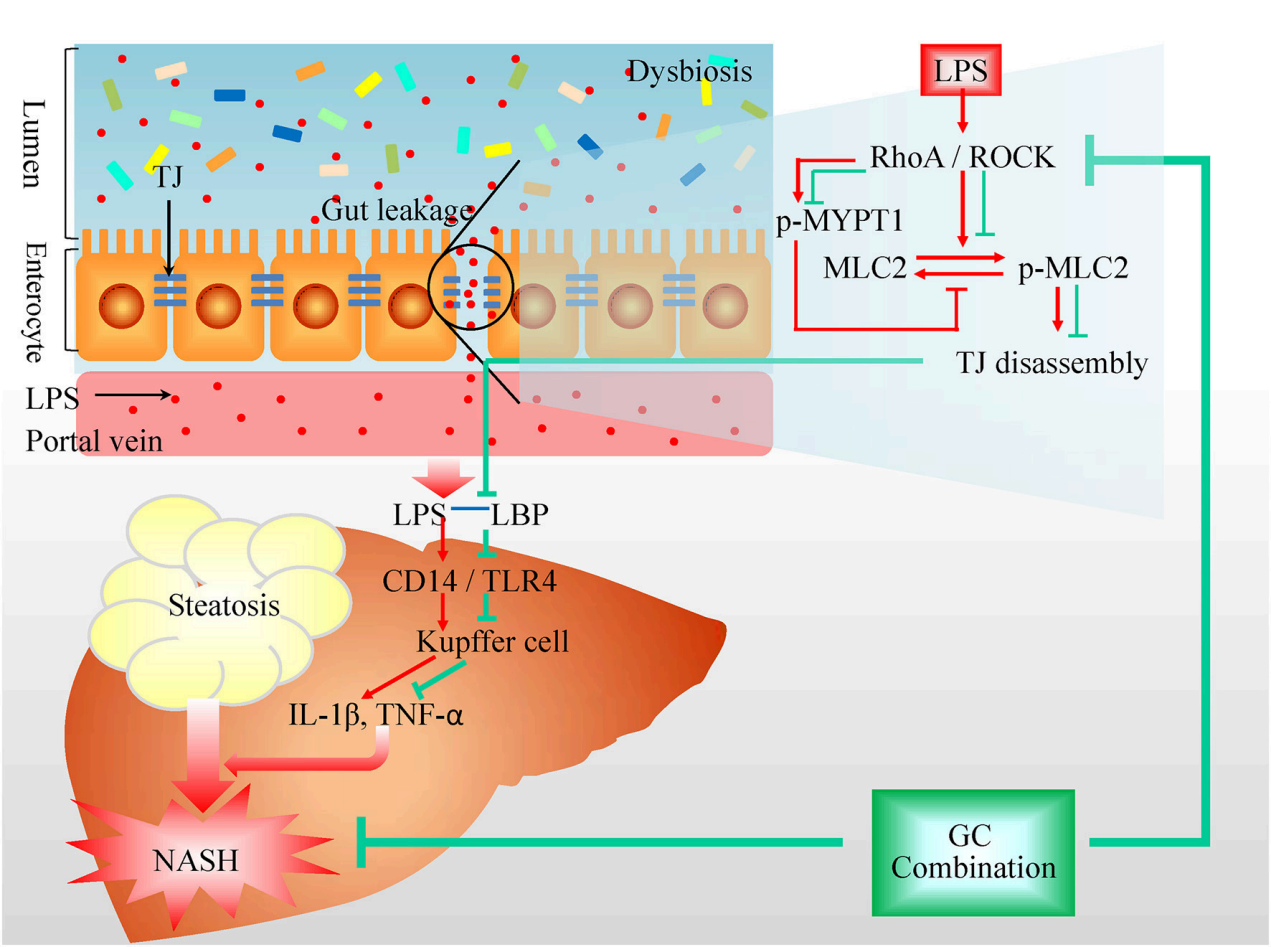
Protection on intestinal barrier function contributes to the therapeutic effects of GC combination on NASH. Overgrowth of the Gram-negative bacteria leads to increased content of LPS in the intestine and gut barrier dysfunction. LPS activates RhoA/ROCK signaling which induces activation of MLC2 via direct phosphorylation or phosphorylation of MYPT. The increased phosphorylation of MLC2 leads to the rearrangement of TJ proteins and increase of TJ permeability. The gut-derived LPS go into liver via portal circulation and activate KCs to produce inflammatory factors participating in the onset of NASH. GC combination was found to restore the TJs protein expression and down-regulate RhoA/ROCK signaling while ameliorated NASH. GC, Geniposide and chlorogenic acid combination, IL-1β, interleukin-1β, LBP, lipopolysaccharide binding protein, LPS, lipopolysaccharide, MLC, myosin light chain, MYPT, myosin phosphatase target subunit, NASH, non-alcoholic steatohepatitis, ROCK, Rho-associated kinase, TNF, tumor necrosis factor, TLR, Toll-like receptor, TJ, tight junction.

In GC-treated mice, the pathological changes in liver tissue, hepatic lipid deposition, and ALT in plasma were all ameliorated, which was comparable to that of QHD-treated mice. Furthermore, plasma LPS level, hepatic infiltration of KCs, LBP content and TLR4 protein expression, as well as the proinflammatory cytokines were all decreased with GC or QHD treatment comparing to that in HFD group. With these data, GC combination was confirmed as the effective components formula in QHD, and inhibition on LPS signaling mediated by KCs contributed to the therapeutic effects of GC and QHD on NAFLD (Figure 8).

### Gut Barrier Dysfunction in NAFLD

In the group of positive control of intestinal mucosa protectant (NaB), the pathological changes of NAFLD mentioned above were all alleviated, which suggested the important role of gut barrier function in NAFLD. Coupled with our previous founds that dysbiosis of gut microbiota existed in NAFLD induced by HFD, especially in the increased abundance of genera Escherichia/Shigella, containing opportunistic pathogens, the main source of gut-derived LPS (Yin et al., 2013), it is presumable that the increased LPS level in plasma probably due to gut barrier dysfunction. Hence, we detected the other biomarker of gut-leakage, D-lactate (Peoc'h et al., 2018), the product of anaerobic glycolysis by microorganisms in the gastrointestinal system. The level of D-lactate in plasma was also increased in the HFD-feeding mice, which confirmed the increased intestinal permeability in HFD group. The intestinal permeability is regulated by TJs, the proteins occupying the paracellular position among adjacent enterocytes, allowing the passage of ions and small molecules and blocking the passage of micro-organism in intestine to liver (Federico et al., 2016). In the present study, the protein expression of colonic TJs, ZO-1 and Occludin, were disrupted in HFD-feeding mice. With treatment of GC combination or QHD, the level of LPS and D-lactate in plasma decreased and protein expression of ZO-1 and Occludin was restored comparing to that in HFD group. These data demonstrated that protection on the intestinal epithelial barrier to prevent the damage induced by gut-derived LPS in liver contributed to the therapeutic effects of GC and QHD on NAFLD.

### RhoA Signaling in Intestinal Epithelial TJs Regulation

The mechanism by which epithelial TJs are disassembled involves the phosphorylation at serine 19 of MLC2, leading to the rearrangement of TJ proteins and increase of TJ permeability (Shen et al., 2006). It is reported that MLC2 phosphorylation is mediated by myosin light chain kinase (MLCK)-dependent or MLCK-independent manner. Among the multiple signaling pathways that regulate TJs, LPS activates RhoA signaling via inducible nitric oxide synthase (Wu et al., 2011). RhoA is a member of Rho family, belonging to the Ras superfamily of small GTP binding proteins and has been reveal to disrupt the barrier function of TJs (Jou et al., 1998). ROCKs, the downstream effectors of RhoA, stimulate the activation of myosin by phosphorylation of MLC2 in a MLCK-independent pathway (Amano et al., 1996; González-Mariscal et al., 2008). On the other hand, ROCKs also phosphorylate and inactivate MYPT, leading to inactivation of the phosphatase and, hence, increased phosphorylation of MLC2 (Essler et al., 1998). In the present study, besides the decreased protein expression of colonic TJs (ZO-1, Occludin), the phosphorylation of MYPT1 at Thr696 and MLC2 at Ser19 were both up-regulated in the colon of HFD-feeding mice, indicating the TJs disassembled. Furthermore, the ROCK activity and the ratio of active RhoA in colon tissue were both increased in HFD group. With treatment of GC combination or QHD, the protein expression of phosphorylated MYPT1 and MLC2 were both down-regulated, as well as ROCK activity and the ratio of active RhoA in colon tissue. These data suggested that GC combination and QHD regulated TJs in ROCK/RhoA-dependent way (Figure 8).

In NaB group, as expected, the level of D-lactate and LPS in plasma decreased, the protein expression of TJs (ZO-1, Occludin) was restored and the phosphorylation of MLC2 and MYPT1 were down-regulated comparing to that in HFD group. While, no effects of NaB on ROCK activity and the ratio of active RhoA were observed, which indicated that NaB regulated intestinal TJs probably in a RhoA-independent way.

### Butyrate as a Protectant of Intestinal Barrier Function

Butyrate is one of the most abundant short-chain fatty acids in the gastrointestinal tract, which is produced by bacterial fermentation of undigested dietary carbohydrates (Bedford and Gong, 2018) and plays an important role in the maintenance of the gut barrier function. Butyrate protects the intestinal epithelium by stimulation the synthesis of mucin 2 and trefoil factor 3 in colon in mice (Song et al., 2006; Bai et al., 2010; Hudcovic et al., 2012) and facilitating the assembly of TJs in the Caco-2 cell and cdx2-IEC monolayer (Peng et al., 2007; Peng L. et al., 2009; Wang et al., 2012). Butyrate reduces bacterial translocation in human colon-derived T84 or HT-29 epithelial cell lines exposed to dinitrophenol and Escherichia coli (Lewis et al., 2010).

As free butyrate is largely absorbed in the upper gastrointestinal tract (Pituch et al., 2013), butyrate salts, butyrate glycerides, and different encapsulation techniques are developed to prevent the release of butyrate in the upper gastrointestinal tract. NaB administrated by gavage has been demonstrated to improve gut microbiota and gastrointestinal barrier in steatohepatitis induced by high-fat diet (HFD) (Zhou et al., 2017) or methionine-choline-deficient diet (Ye et al., 2018). Furthermore, NaB promotes reassembly of TJs in Caco-2 monolayer involving inhibition of MLCK/MLC2 pathway and phosphorylation of protein kinase C β2 (Miao et al., 2016).

Consistently, in the present study, NaB was observed to restore the TJs and inhibit gut-derived LPS leakage. Since it is well accepted that the impaired gut barrier function contributes to the pathogenesis of NAFLD, we believe that restoration of intestinal barrier function by NaB contributes to the amelioration of NAFLD although it has other pharmacological effects, such as action as a histone deacetylase, anti-oxidation, anti-inflammation, and so on.

### Protection on Gut Barrier Is Not the Exclusive Action Mechanism of GC Combination on NAFLD

Actually, our previous studies have demonstrated that GC (Feng et al., 2017) and QHD (Yin et al., 2013) treat NAFLD and modulate the structure of the gut microbiome simultaneously. While GC combination treatment did not decrease the abundance of Gram-negative bacteria, the source of gut-derived LPS (Feng et al., 2017), suggesting that the compositional change in the microbiota is not the cause of decreased LPS in plasma by GC. The results of the present study demonstrated that restored function of TJs by GC combination prevents gut-derived LPS leakage from intestine, which probably contributed to the therapeutic effects of GC on NAFLD. The protective effects of GC combination on gut barrier in NAFLD mice are correlated closely to restoration of the protein expression of TJs and inhibition on TJs disassembly by down-regulation of RhoA/ROCK signaling (Figure 8).

The effects of geniposide and chlorogenic acid on gut were reported recently. Geniposide ameliorates inflammation and mucosal damage in colitis (Xu et al., 2017; Zhang et al., 2017a), which is correlated to regulation on nuclear factor kappa B, peroxisome proliferator-activated receptor γ and MCL2 pathways and the expressions of ZO-1 and occludin. Chlorogenic acid was reported to preserve intestinal morphological integrity, regulate intestinal microbiota, improve antioxidant capacity, suppress mucosa inflammation and cell apoptosis in weaned piglets (Chen et al., 2018a,b; Zhang et al., 2018) and in mice with colitis (Zhang et al., 2017b). In our further study, it is deserved to be investigated that the individual role of geniposide and chlorogenic acid plays in the effects of GC combination on gut barrier function in NAFLD.

On the other hand, geniposide and chlorogenic acid do have effects on liver in NAFLD. Geniposide was reported to alleviate lipid accumulation in liver of obese and type 2 diabetic mice and HepG2 cells treated with free fatty acid (Kojima et al., 2011), inhibit inflammation by suppressing methyl cytosine binding protein-2 in acute liver injury induced by carbon tetrachloride in mice (Ma T. T. et al., 2015). Chlorogenic acid was reported to curb hepatic steatosis, which correlated to suppression on hepatic gene expression of peroxisome proliferator-activated receptor γ, CD36, and fatty acid binding protein 4 induced by HFD (Ma Y. et al., 2015). Chlorogenic acid was also demonstrated to ameliorate hepatic lipid accumulation by regulation on the cholesterol metabolism in hepatocyte (Hao et al., 2016).

Based on the results obtained by our studies and the previous reports, we believe that the action mechanism of GC combination on NAFLD is multi-targeting. Besides intestine, the effects of GC combination on liver are also deserved thorough investigation.

Combination composed of more than one chemical with clear mechanisms of action and safety data has been accepted to treat the diseases with complicated pathogenesis mechanisms, such as fixed-dose combinations of metformin and other diabetes drugs in type 2 diabetes treatment (American Diabetes Association, 2018) and Sacubitril-Valsartan in heart failure treatment (Ollendorf et al., 2016). We think the presented work will help us to understand the mechanisms and the responsible components of the therapeutic effects of traditional Chinese medicine on NAFLD and probably provide a very preliminary clue to develop some fixed-dose combination composed of chemical molecules with understood structures and action modes from the traditional Chinese medicine to treat NAFLD.

## Author Contributions

JP and YH designed the research. JP, JL, HT, YF, QF, and YZ performed research. TY performed UHPLC-MS analysis. JP analyzed data and wrote the paper.

### Conflict of Interest Statement

The authors declare that the research was conducted in the absence of any commercial or financial relationships that could be construed as a potential conflict of interest.

## Abbreviations

ALT: alanine aminotransferase
ANOVA: one-way analysis of variance
DDW: double distilled water
ELISA: enzyme linked immunosorbent assay
GC: Geniposide and chlorogenic acid combination
H&E: hematoxylin and eosin
HFD: high fat diet
IL: interleukin
LBP: lipopolysaccharide binding protein
LPS: lipopolysaccharide
MLC: myosin light chain
MLCK: myosin light chain kinase
MYPT: myosin phosphatase target subunit
MyD88: myeloid differentiation factor 88
NAFLD: non-alcoholic fatty liver disease
NASH: non-alcoholic steatohepatitis
NAS: NAFLD activity score
NaB: sodium butyrate
OCT: optimal cutting temperature medium
QHD: Qushi Huayu Decoction
ROCK: Rho-associated kinase
TG: triglyceride
TJ: tight junction
TNF: tumor necrosis factor
TLR: Toll-like receptor
UHPLC-MS: ultra-high performance liquid chromatography-mass spectrometry
ZO-1: zonula occludens-1.
