# Supplementary material for: Geniposide and Chlorogenic Acid Combination Ameliorates Non-alcoholic Steatohepatitis Involving the Protection on the Gut Barrier Function in Mouse Induced by High-Fat Diet
Source: Front Pharmacol. 2018 Dec 11;9:1399. doi: 10.3389/fphar.2018.01399 (PMC6298419; doi:10.3389/fphar.2018.01399)
Supplement: Supplementary file 1 [file Image_1.pdf]

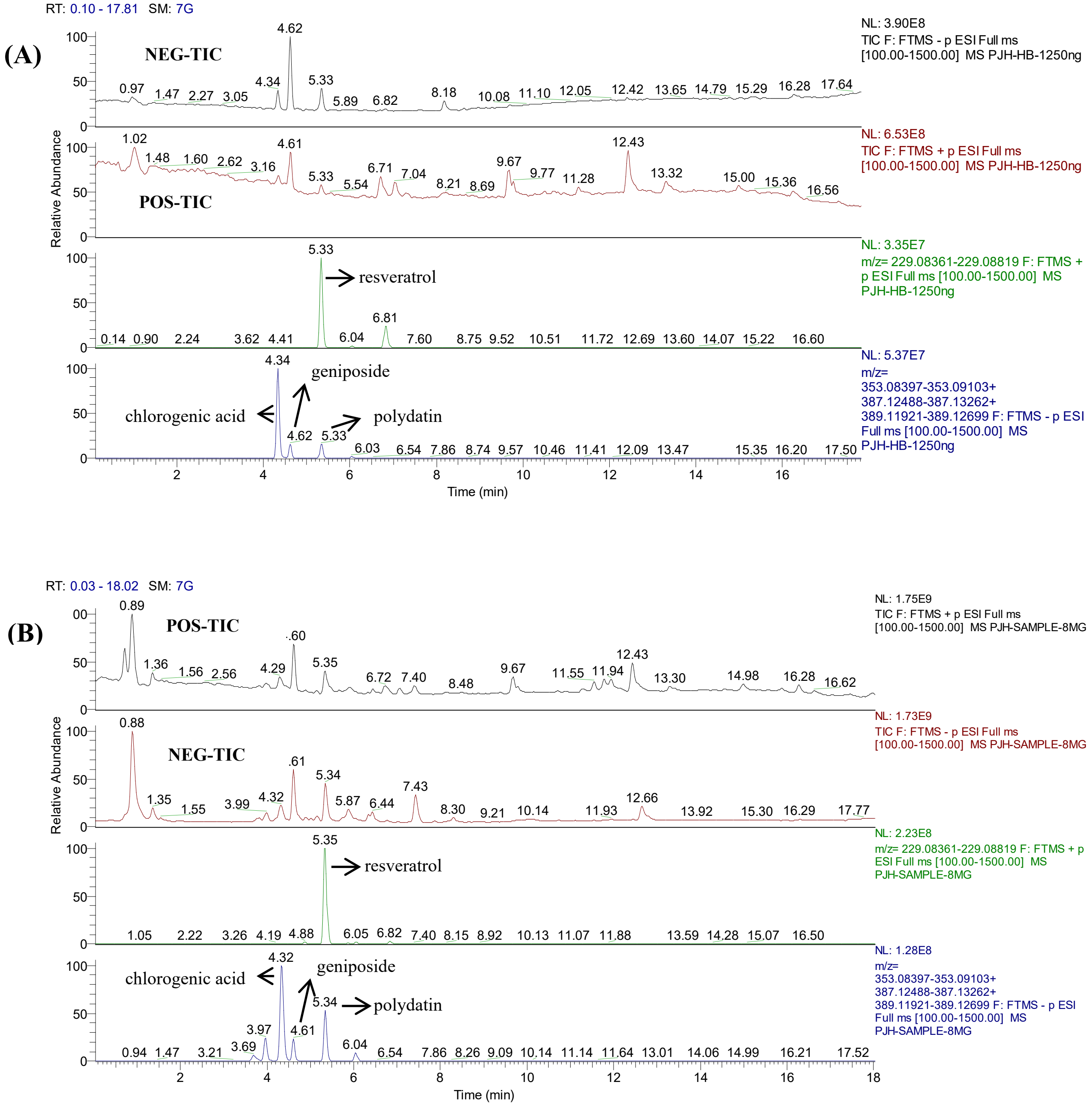

Supplementary figure 1. Total-ion chromatograms (TIC) and select-ion chromatograms of Qushi Huayu Decoction (QHD). (A) the mix standard solution, 1.25ug/ml; (B) QHD solution. Stationary phase: ACQUITY UPLC HSS T_3 (2.1 mm×100 mm, 1.8 μm); mobile phase: acetonitrile (I) and water (II) in gradient (time, min/II%: 0/95, 18/5); flow rate: 0.3 mL/min). Retention time: 4.32 min, chlorogenic acid $[M-1]^{-}$, 4.61 min, geniposide $[M-1]^{-}$, 5.34 min, polydatin $[M-1]^{-}$, 5.33 min, resveratrol$[M+1]^{+}$.
